# Altered Progesterone Receptor Isoform Expression in Endometrial Cells: A Preliminary Immunohistochemical Study in Endometriosis

**DOI:** 10.3390/jcm15176664

**Published:** 2026-08-28

**Authors:** Kathryn Coyne, Chloe Van Dorn, Sung Tae Kim, Joseph Findley, Rebecca Flyckt, Sam Mesiano, Rachel Weinerman

**Affiliations:** 1 Division of Reproductive Endocrinology and Infertility, Department of Obstetrics and Gynecology, University Hospitals Cleveland Medical Center, Cleveland, OH 44106, USA; sungtae.kim@uhhospitals.org (S.T.K.); joseph.findley@uhhospitals.org (J.F.); rebecca.flyckt2@uhhospitals.org (R.F.); rachel.weinerman@uhhospitals.org (R.W.); 2 School of Medicine, Case Western Reserve University, Cleveland, OH 44106, USA; 3 Department of Reproductive Biology, Case Western Reserve University, Cleveland, OH 44106, USA; sxm188@case.edu

**Keywords:** endometriosis, endometrium, progesterone receptor, phosphorylation, infertility

## Abstract

**Background/Objectives:** The objective of this exploratory study was to test the hypothesis that endometriosis is associated with altered abundance and cellular location of nuclear progesterone receptor (PR) isoforms in eutopic endometrium. **Methods**: Immunohistochemistry (IHC) was used to compare total PR (PR-A/B), PR-B, and pSer344/345-PR staining in endometrium from patients with and without endometriosis and between eutopic endometrium and ectopic endometriotic lesions in patients with endometriosis. Reproductive-age female patients undergoing laparoscopy for endometriosis (*n* = 10), or for other indications and without a diagnosis of endometriosis (*n* = 10), were included in this case–control study. **Results:** Eutopic endometrium from patients with endometriosis demonstrated increased total PR (PR-A/B) staining relative to PR-B staining compared with patients without endometriosis; a similar staining pattern was observed in ectopic lesions. Because PR-A was not measured directly and the total PR and PR-B antibodies may differ in affinity and dynamic range, these findings provide indirect evidence of altered relative PR isoform abundance rather than direct demonstration of a PR-A-dominant state. pSer344/345-PR staining did not differ significantly between groups, although a nonsignificant trend toward higher staining was observed in endometriosis. **Conclusions:** These preliminary findings support further study of PR isoform balance and site-specific phosphorylation in progesterone resistance associated with endometriosis.

## 1. Introduction

Endometriosis, a disease whereby endometrial cells implant in ectopic locations outside of the endometrium, negatively affects fertility and pregnancy, and the hormones produced by the ovary, progesterone and estradiol, significantly impact the development and progression of the disease. During the normal menstrual cycle, progesterone promotes differentiation of endometrial epithelial and stromal cells [1,2,3] and increases the secretory activity of the epithelium and induces decidualization in the stroma [4]. Progesterone acts as an antiestrogen through paracrine signaling, leading to the conversion of estradiol into the less potent estrogen, estrone [5].

While patients with endometriosis exhibit circulating progesterone levels comparable to those without the condition, ectopic lesions in endometriosis do not respond appropriately to progesterone [6]. The concept of progesterone resistance emerged from studies indicating that progesterone treatment failed to convert estradiol to the less potent estrone [7]. Furthermore, there is a reduction in progesterone-induced prolactin production in ectopic endometrium, providing additional evidence of progesterone resistance [8]. Altered signaling of these hormones, particularly progesterone, has been associated with increased risk of eutopic endometrial cells forming ectopic implants of endometrium, which is theorized to occur via retrograde menstruation [9]. Therefore, altered responsiveness of endometrial cells to progesterone may promote the development of endometriosis.

Progesterone affects the endometrium through progesterone receptor (PR) isoforms PR-A and PR-B, which are expressed in both endometrial stromal and glandular epithelial cells. Stromal PR signaling is particularly important in mediating paracrine progesterone effects on endometrial epithelial function. PR-B is the full-length isoform, whereas PR-A lacks the first 164 amino acid residues of PR-B [10,11]. These isoforms function as ligand-activated transcription factors. In many experimental systems, PR-B has stronger progesterone-induced transcriptional activity than PR-A, and PR-A can repress PR-B transcriptional activity in a cell- and promoter-dependent manner [12]. Initial studies demonstrated that PR-B can act as a strong transcriptional activator in response to progesterone, whereas PR-A is less active and, in several contexts, inhibits PR-B when its abundance exceeds that of PR-B [12,13,14,15,16,17].

It is these initial observations that led to the concept that PR-A and PR-B have opposing transcriptional activity, and as such progesterone responsiveness is inversely related to the PR-A:PR-B ratio. This mechanism allows the target cell control of progesterone responsiveness via modulation of isoform levels, and it has been hypothesized that this inverse relationship applies to endometrial function in endometriosis. An abnormal expression of the two PR isoforms may provide an explanation for the altered response to progesterone and especially progesterone resistance.

The levels of PR-A and PR-B in stromal compared to glandular cells of the endometrium are variable throughout phases of the menstrual cycle. In the normal menstrual cycle, the levels of both PR-A and PR-B exhibit a progressive increase during the proliferative phase and subsequently decrease after ovulation, indicating that estradiol stimulates the production of progesterone receptors. However, in endometriotic tissues, this pattern is not observed [18]. Attia and colleagues, using Western blot, reported decreased protein levels of PR-B compared to PR-A in endometriotic lesions, and then using RNase protection assay also demonstrated indirectly that only PR-A transcripts were present in endometriotic lesions [9].

These findings supported the concept that progesterone responsiveness is inversely related to the level of PR-A relative to PR-B. Importantly, anti-inflammatory actions mediated via PR-B would be decreased in cells where PR-A dominates. Indeed, further studies have shown that endometriosis was associated with increased abundance of PR-A relative to PR-B in eutopic uterine endometrium [19]. Bedaiwy et al. found that endometriotic lesions may be derived from eutopic endometrium with aberrant PR responsiveness, and have been associated with alterations in the relative abundance of the PR isoforms [19]. Moreover, it has been shown that there are reduced levels of progesterone receptors in endometriotic tissue compared to normal endometrium [20,21,22,23,24]. This implies that progesterone resistance is, to some extent, influenced by the decreased levels of PR [9,25,26], and suggests that the eutopic endometrium of patients with endometriosis, along with ectopic endometrial implants, may also be progesterone resistant.

Dysfunction of progesterone action on endometrial cells due to aberrant PR abundance could lead to the downstream effects of endometriosis, and subsequently infertility and pregnancy loss. Further investigation has revealed various potential mechanisms underlying progesterone resistance. As mentioned previously, endometriosis is characterized by a chronic inflammatory state, and progesterone typically serves as an anti-inflammatory agent in uterine cells. In the context of endometriosis, an influx of proinflammatory cytokines occurs. This proinflammatory environment could negatively impact PR function by influencing chaperone proteins, receptor coregulators, and progesterone receptor gene expression [27].

More recent studies indicate that both PR isoforms are transcriptionally active at diverse sets of endogenous promoters and that the cellular response to progesterone reflects the combined, context-dependent actions of PR-A and PR-B [15,28]. The ability of PR isoforms to target distinct promoters and regulate downstream genes is influenced by cell type, transcriptional coregulators, interactions with other transcription factors, and post-translational modifications including serine phosphorylation [28,29].

In particular, site-specific serine phosphorylation (pSer) can modulate PR transcriptional activity and progesterone responsiveness. Multiple serine residues within the amino-terminal domain are phosphorylated; Ser81, Ser102, and Ser162 are unique to full-length PR-B, whereas Ser190, Ser294, Ser345, and Ser400 are shared by both isoforms [30]. Phosphorylation at these sites can affect receptor stability, hormone sensitivity, nuclear localization, and promoter targeting [28,29,30]. We focused on pSer344/345-PR because prior work using the same site-specific antibody demonstrated robust detection of this phospho-PR form in uterine myometrial cells and showed that phosphorylation at Ser344/345 can modify PR-A functional activity in that tissue [31]. Much of the foundational work on site-specific PR phosphorylation has been performed in breast cancer models, while uterine studies support tissue-specific functional relevance of these conserved phosphorylation sites [28,29,30,31].

PRs have been shown to be key mediators of progesterone action in uterine tissues, both endometrial and myometrial tissues, and are essential for normal uterine function [28]. In preliminary studies, pSer^344/345^-PR was detected in uterine myometrial cells, suggesting that progesterone modulation of the myometrium involves generation of this pSer-PR [31]. This study demonstrates that within myometrial cells, pSer345 activates the inhibitory capacity of PR-A on anti-inflammatory actions of progesterone mediated by PR-B [31]. Phosphorylation at this site thus appears to alter PR-A activity leading to a decrease in net progesterone responsiveness, further implicating a role for epigenetic modifications in mediating progesterone resistance. Whether pSer344/345-PR is involved in the etiology of endometriosis, possibly via its aberrant generation in eutopic or ectopic endometrium, is unknown. Direct mechanistic links between PR-A phosphorylation and epigenetic changes in endometriosis are not fully elucidated, but it is possible that PR-A phosphorylation may alter its transcriptional activity through changes in co-regulator binding or chromatin recruitment. It may also influence epigenetic regulators indirectly via kinase signaling pathways (e.g., AKT/ERK), contributing to PR silencing and hormonal resistance.

The objective of this exploratory study was to evaluate the abundance and cellular location of total PR (PR-A/B), PR-B, and pSer344/345-PR in eutopic and ectopic endometrium obtained from patients with and without clinical endometriosis. We hypothesized that endometriosis would be associated with altered relative PR immunostaining and potentially altered pSer344/345-PR staining. Because PR-A was not measured directly, comparisons of total PR and PR-B staining were interpreted as indirect evidence of relative isoform imbalance rather than a direct measure of the PR-A:PR-B ratio.

## 2. Materials and Methods

### 2.1. Patients

The study received approval by the institutional review board (IRB) for human research and was conducted between May 2022 and December 2023 (IRB study number 20220053). Patients eligible for inclusion were female, age 18–45 years old, with regular menstrual cycles lasting between 25 and 35 days and no hormonal treatments to modify fertility or menstrual cycles for a minimum of 3 months before the surgery, and those with the ability to provide informed consent and authorization. Exclusion criteria included patients with concern for hyperplasia or cancer on surgical or endometrial pathology. All patients recruited underwent pre-operative ultrasounds to evaluate for potential sonographic signs of endometriosis and or intrauterine pathology. Additionally, all patients received pre-operative counseling and informed consent was obtained prior to the day of surgery.

### 2.2. Sampling

Based on prior quantitative immunoblot data [19], an initial sample size of 10 per clinical group was estimated to provide 80% power to detect a two-fold difference in mean PR levels. That calculation assumed quantitative relative optical-density measurements and is not directly transferable to the ordinal 0–3 semi-quantitative IHC outcome used as the primary measure in this study. The present analysis is therefore considered exploratory, and the phase-stratified comparisons (*n* = 4–6 per subgroup) are hypothesis-generating.

### 2.3. Data Collection

At the time of surgery, biopsies of eutopic endometrium and peritoneal endometriosis were obtained synchronously from the same patients undergoing operative laparoscopy for high clinical suspicion for or known history of endometriosis (*n* = 10). Histopathology confirmed endometriosis; two of these 10 patients had deep endometriosis. Detailed rASRM stage and lesion phenotype were not uniformly recorded for all participants. Biopsies of endometrium and disease-free peritoneum were also obtained synchronously from patients without laparoscopic evidence of endometriosis undergoing laparoscopy for other infertility-related indications (*n* = 10), including myomectomy and diagnostic laparoscopy for unexplained infertility. All participants had infertility. Tissue samples were stratified by menstrual-cycle phase at collection, determined from the last menstrual period and confirmed by endometrial histology; the endometriosis group included five proliferative and five secretory samples, and the control group included four proliferative and six secretory samples. An independent pathologist reviewed all tissue samples, confirmed menstrual phase, and excluded other endometrial pathology such as hyperplasia. Each patient contributed only one sample per tissue type. Participants had not used hormonal treatments to modify fertility or menstrual cycles for at least three months before surgery; more remote treatment history and infertility etiology were not systematically characterized for all participants.

Each specimen was washed several times in ice-cold phosphate-buffered saline to remove blood and extraneous tissue, such as pre-peritoneal adipose tissue, and then was formalin fixed for histological analysis and immunohistochemistry. The tissues fixed in formalin were embedded in paraffin, and sections of 5 µm thickness were cut and mounted on glass slides. Menstrual cycle stage was determined by histopathological examination.

### 2.4. Immunohistochemistry

Immunohistochemistry was utilized to determine the cellular and sub-cellular location of PR-B, total PR (PR-A/B), and pSer344/345-PR. Tissue sections were deparaffinized in xylene, rehydrated in graded ethanols, and subjected to heat-activated antigen retrieval at 95 °C in 10 mmol/L citric acid, 0.05% Tween 20, pH 6.0 for 20 min. Sections were then incubated in blocking buffer (VECTASTAIN ABC-HRP Kit; Vector Laboratories Inc., Burlingame, CA, USA) at room temperature and then rinsed in Tris-buffered saline with 0.1% Tween 20 detergent (TBST). Primary antibodies anti-PR-B (rabbit monoclonal; Cell Signaling Technology, Danvers, MA, USA; cat. #3157S), anti-total PR/PR-A/B (rabbit monoclonal; Cell Signaling Technology, Danvers, MA, USA; cat. #8757), and anti-pSer344/345-PR (rabbit monoclonal; Cell Signaling Technology, Danvers, MA, USA; cat. #12783), each diluted 1:250 in SignalStain^®^ Antibody Diluent (8112L), were applied and sections incubated for 30 min at room temperature. Sections were rinsed with TBST and secondary antibody (SignalStain^®^ HRP, Rab 8114S) was applied for 30 min at room temperature. Sections were then rinsed in TBST and bound immunoreactive signal was detected using a peroxidase/3,3′-diaminobenzidine-based visualization kit (DAB Substrate Kit, Peroxidase [HRP], with Nickel, SK-4100; Vector Laboratories Inc., Burlingame, CA, USA), counterstained with hematoxylin, and processed for coverslip application.

IHC staining intensity and cellular localization were evaluated under light microscopy. Two independent observers, blinded to sample identity, scored staining intensity using the method outlined by Mote et al. on an ordinal scale from 0 to 3 (0 = no staining, 1 = mild, 2 = moderate, 3 = strong) [32]. The scores assigned by the two observers were averaged to generate the final staining-intensity score used for statistical analysis. Interobserver agreement was assessed using a two-way, single-measure, absolute-agreement intraclass correlation coefficient (ICC). Agreement between observers was excellent (ICC = 0.952; 95% CI, 0.922–0.972).

### 2.5. Statistical Analysis

Because the IHC staining score is ordinal and the cohort is small, analyses are considered exploratory. Between-group comparisons of independent samples were evaluated using nonparametric methods where appropriate. Menstrual-phase and glandular/stromal subgroup analyses were exploratory because of small cell sizes (*n* = 4–6) and no correction for multiple comparisons was applied. Semi-quantitative IHC data were analyzed using nonparametric methods given the ordinal nature of the staining-intensity scores and small sample size. Comparisons between independent endometriosis and control groups were performed using the Mann–Whitney U test. Because eutopic endometrium and ectopic endometriotic lesions were synchronously obtained from the same patients, comparisons between these tissues were performed using paired Wilcoxon signed-rank tests. No formal correction for multiple comparisons was applied given the exploratory nature of the study. Differences were considered statistically significant at *p* < 0.05.

## 3. Results

### 3.1. Patient Characteristics

Patient characteristics between the endometriosis and control groups were similar with the exception of age (Table 1). The mean (±SD) age of the endometriosis group was younger at 31.1 ± 4.2 years compared with 35.7 ± 3.9 years for the control group (*p* = 0.02). Mean body mass index (BMI) was similar at 30.15 ± 4.3 kg/m^2^ and 31.8 ± 6.8 kg/m^2^, respectively (*p* = 0.52). In the endometriosis group, 70% were White, 20% Black, and 10% Asian; in the control group, 70% were White and 30% were Black. All participants had infertility. The age imbalance and the use of an infertile control group are considered important potential sources of confounding in this small exploratory cohort.

### 3.2. PR Immunostaining in Eutopic Endometrium

**Figure 1:**
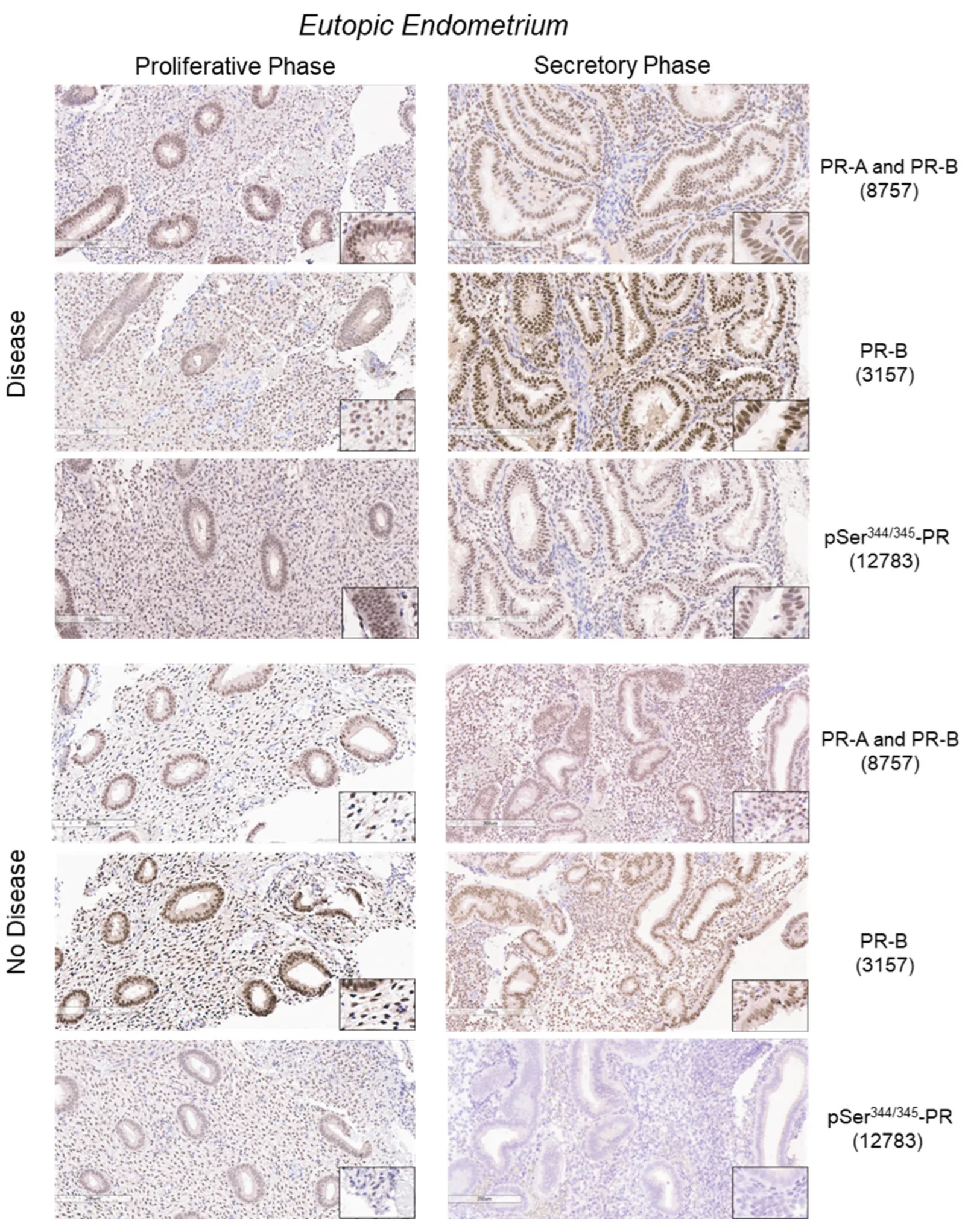
Representative immunostaining of eutopic endometrium in patients with and without endometriosis, shown by menstrual-cycle phase. Antibodies detect total PR (PR-A/B), PR-B, and pSer344/345-PR. PR = progesterone receptor. Representative images are drawn from the study cohort (endometriosis *n* = 10; no endometriosis *n* = 10). Scale bar = 200 um.

**Figure 2:**
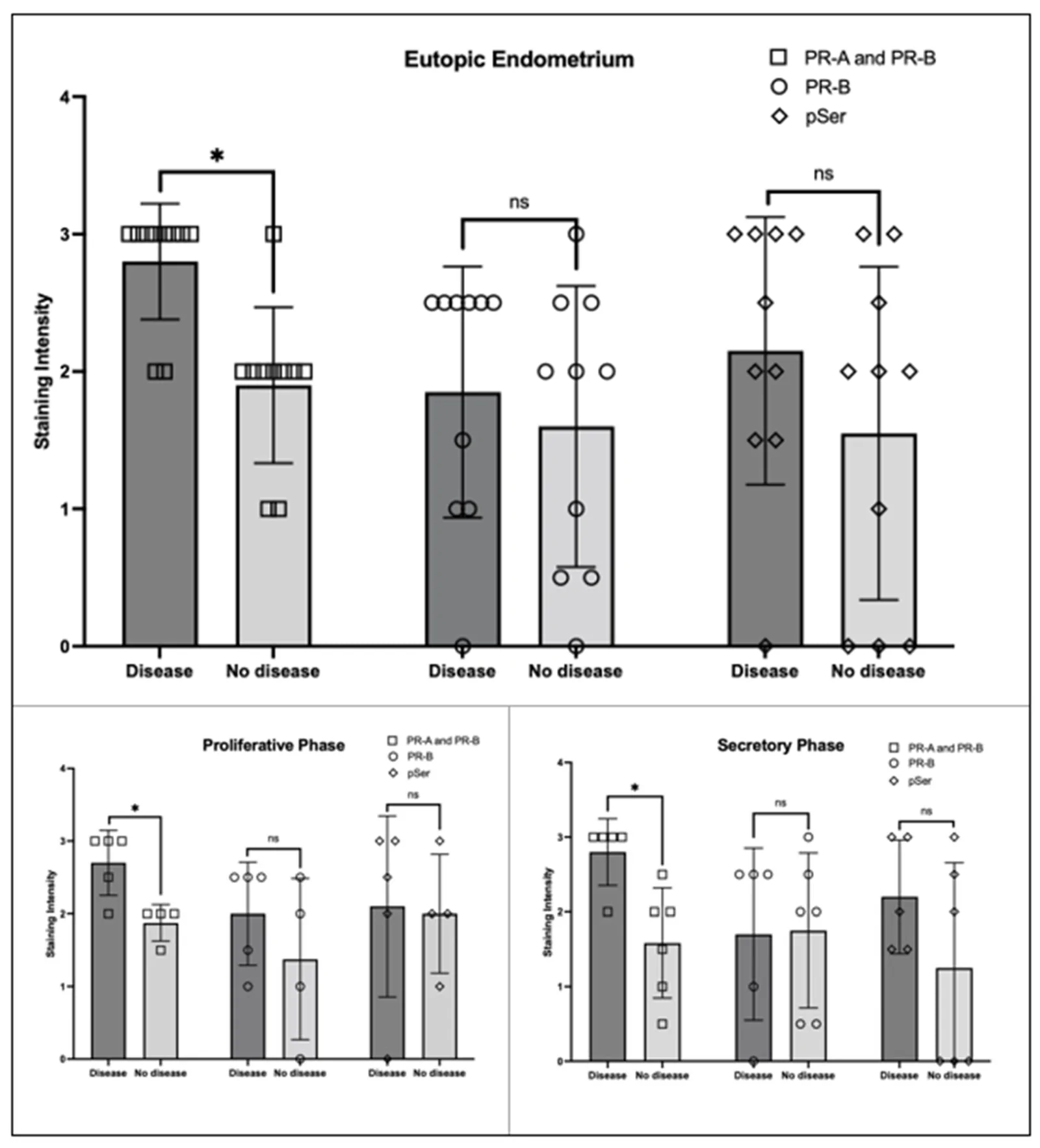
Exploratory analysis of PR staining intensity in eutopic endometrium in patients with (*n* = 10) and without (*n* = 10) endometriosis. Points represent individual samples and bars summarize central tendency with the error bars shown in the source graph. Total PR = PR-A/B; pSer = pSer344/345-PR; ns = not significant; * *p* < 0.05. Because PR staining varies by menstrual-cycle phase and cellular compartment, phase- and compartment-stratified results are provided in Table 2. Statistical comparisons are exploratory and were not adjusted for multiple testing. Values are mean ± SD; error bars indicate SD.

Immunostaining for PR-B, total PR (PR-A/B), and pSer344/345-PR was detected in all endometrial specimens (Figure 1). Mean total PR (PR-A/B) staining intensity was higher in eutopic endometrium from patients with endometriosis compared with patients without disease (2.75 ± 0.42 vs. 1.70 ± 0.59, *p* = 0.0036; Figure 2). This difference was also observed in the phase-stratified exploratory comparisons (proliferative *p* = 0.0397; secretory *p* = 0.0152). Mean staining intensity for PR-B (1.85 ± 0.91 vs. 1.60 ± 1.02, *p* = 0.47) and pSer344/345-PR (2.15 ± 0.97 vs. 1.55 ± 1.21, *p* = 0.28) did not differ significantly between groups. Given the small phase-specific sample sizes and multiple comparisons, these subgroup findings should be interpreted as exploratory.

Immunoreactive staining for PR-B, total PR (PR-A/B), and pSer344/345-PR was nuclear in glandular and stromal cells in both phases of the menstrual cycle (Figure 1). Total PR (PR-A/B) staining was higher in glandular and stromal compartments in women with endometriosis in the phase-stratified comparisons shown in Table 2. No statistically significant differences were identified for PR-B or pSer344/345-PR in glandular or stromal compartments. These analyses are limited by small subgroup sizes (*n* = 4–6 per group/phase) and were not adjusted for multiple testing.

### 3.3. Comparison of Eutopic and Ectopic Endometriotic Tissue

**Figure 3:**
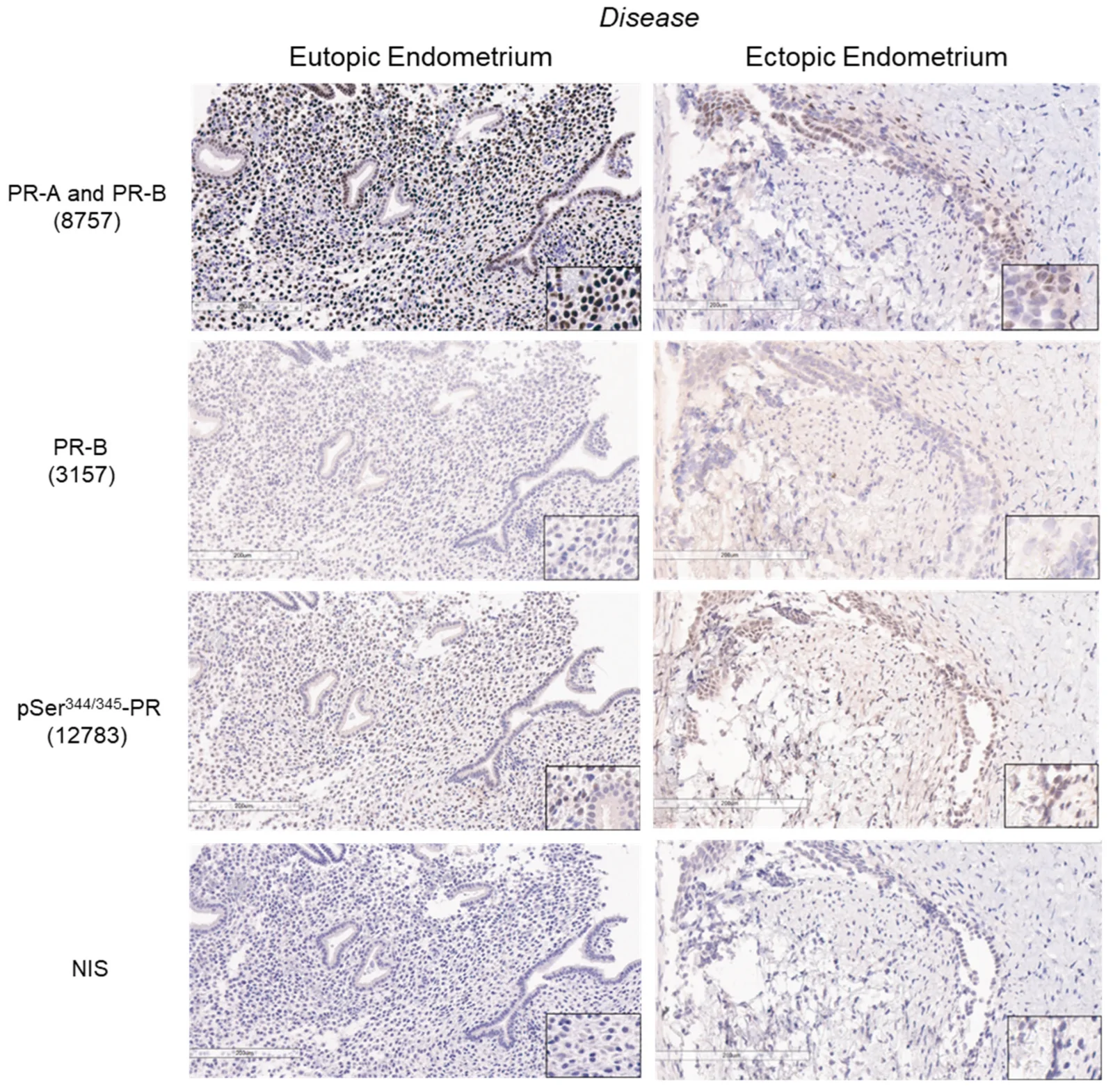
Representative immunostaining of paired eutopic endometrium and ectopic peritoneal endometriotic lesion obtained synchronously from the same patient with endometriosis during the secretory phase of the menstrual cycle. Antibodies detect total PR (PR-A/B), PR-B, and pSer344/345-PR; NIS = non-immune staining. Scale bar = 200 um.

**Figure 4:**
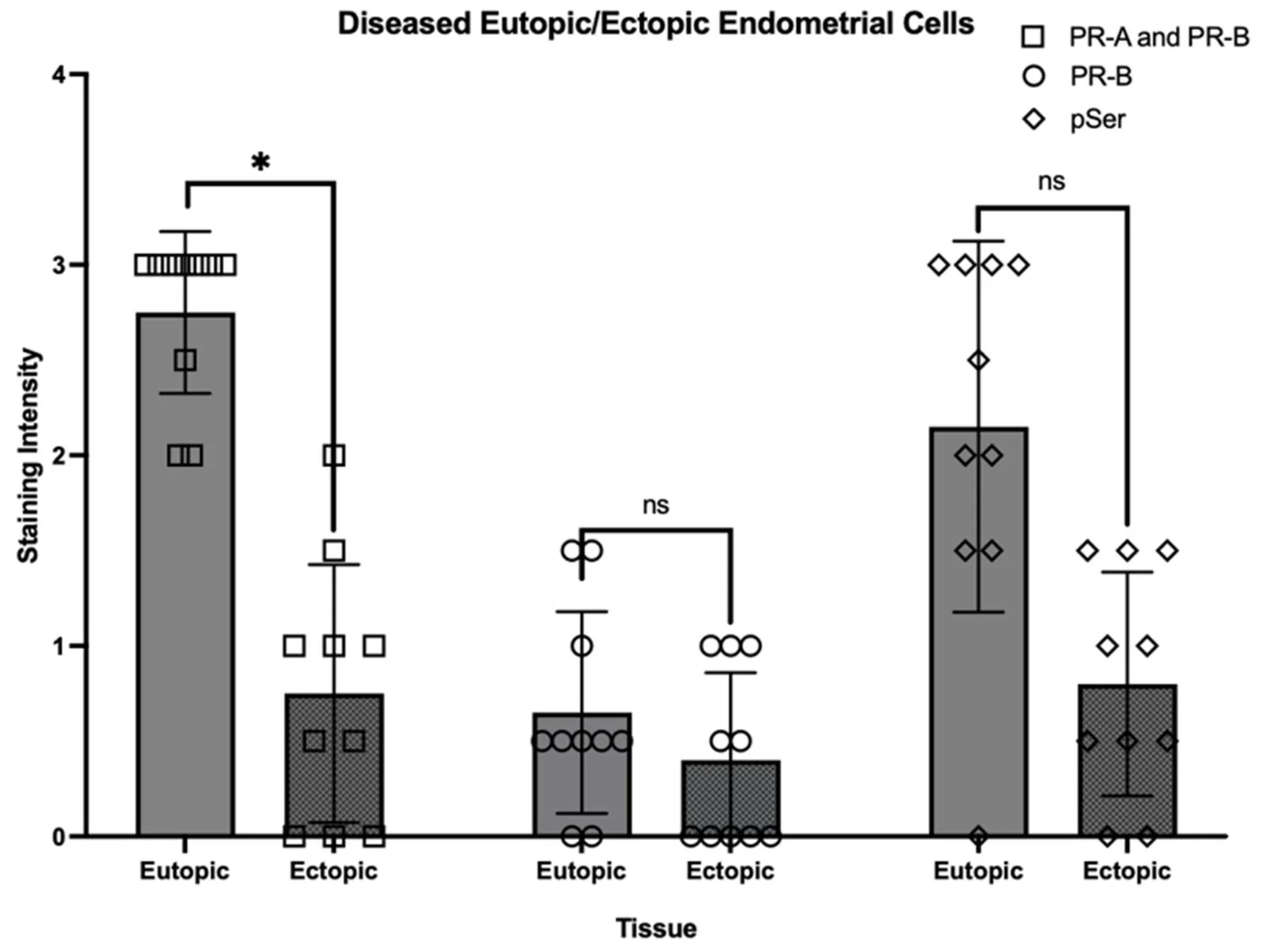
Exploratory comparison of PR staining intensity in paired eutopic endometrium and ectopic peritoneal endometriotic lesions from patients with endometriosis (*n* = 10). Total PR = PR-A/B; pSer = pSer344/345-PR; ns = not significant; * *p* < 0.05. Eutopic and ectopic tissues were collected from the same patient during the same surgery. Points represent individual specimens and bars summarize central tendency with the error bars shown in the source graph. Values are mean ± SD; error bars indicate SD.

In patients with endometriosis, eutopic endometrium and peritoneal endometriotic lesions were obtained synchronously from the same patient during the same surgery and assessed for immunoreactive staining (Figure 3). Given the paired nature of these specimens and the ordinal staining-intensity measure, within-patient comparisons of eutopic and ectopic tissue were performed using paired Wilcoxon signed-rank tests. Staining intensity was significantly greater in eutopic endometrium compared with matched ectopic peritoneal implants for total PR (PR-A/B) (2.75 ± 0.42 vs. 0.75 ± 0.68, *p* = 0.002) and PR-B (1.85 ± 0.91 vs. 0.40 ± 0.46, *p* = 0.008) (Figure 4). No immunostaining was detected in disease-free peritoneum collected from the control group.

**Figure 5:**
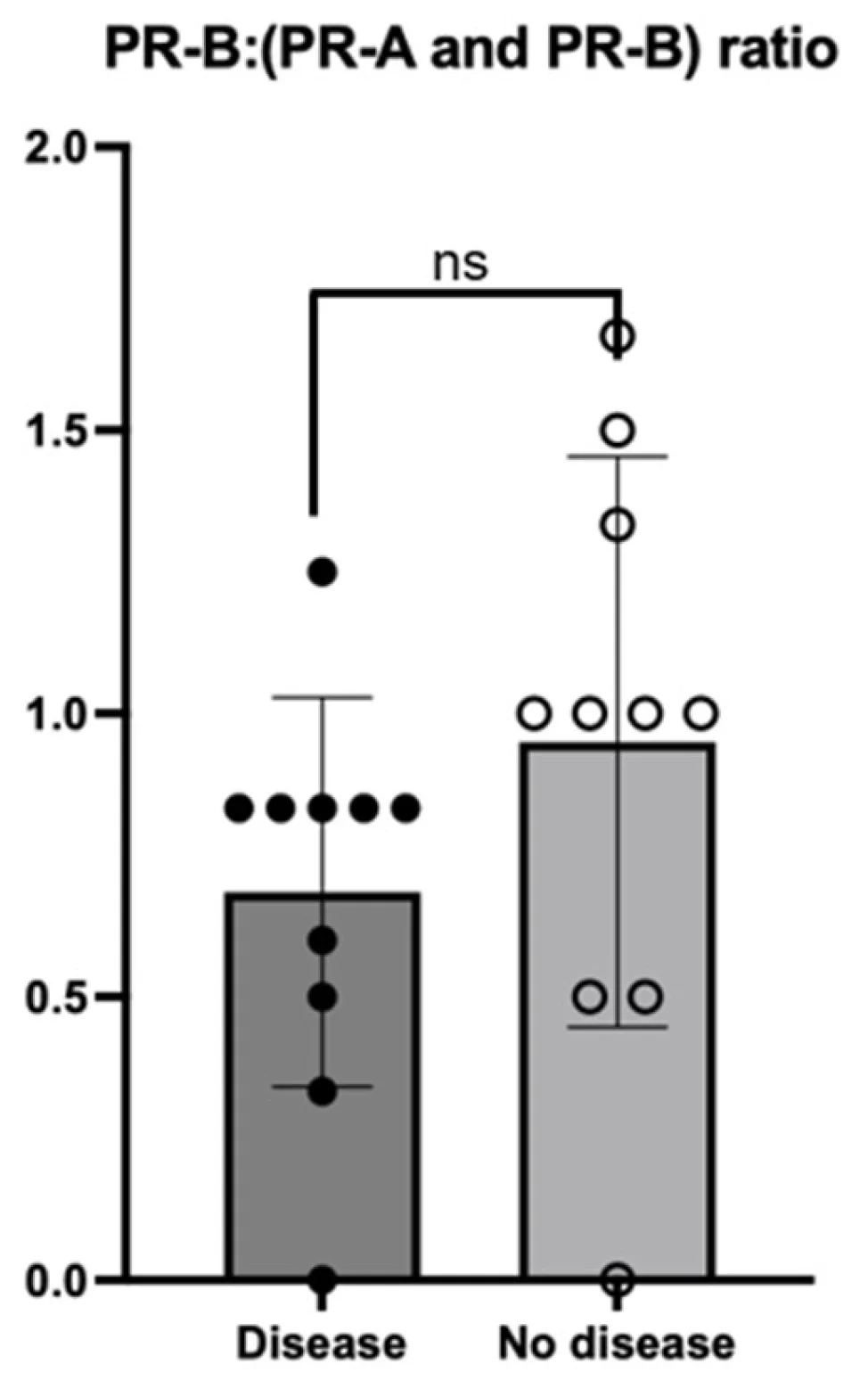
Exploratory PR-B:total PR (PR-A/B) relative-staining index in patients with (*n* = 10) and without (*n* = 10) endometriosis. PR = progesterone receptor; ns = not significant. This index is derived from staining generated by two different antibodies and has not been technically validated as a direct PR-A:PR-B ratio.

The ratio of PR-B staining intensity to total PR (PR-A/B) staining intensity was also explored (Figure 5). This ratio compares signals generated by two different antibodies and has not been technically validated as a quantitative PR-A:PR-B ratio; it is therefore presented only as an exploratory relative-staining index. No statistically significant difference in this index was observed between groups. Because of the small sample sizes within menstrual-cycle phase subgroups, this exploratory analysis was not further stratified by menstrual phase or cellular compartment. Thus, the pooled analysis may obscure phase- or compartment-specific differences and should be interpreted cautiously.

## 4. Discussion

Our findings add to the body of work supporting altered progesterone responsiveness in the pathophysiology of endometriosis. This exploratory study evaluated whether relative PR immunostaining and pSer344/345-PR staining differ in endometriosis. Prior studies have primarily assessed PR-A and PR-B abundance, whereas the present study also examined one site-specific phosphorylated PR form. The principal finding was increased total PR (PR-A/B) staining in eutopic endometrium from patients with endometriosis without a statistically significant difference in PR-B staining. Because PR-A was not directly measured, these data suggest—but do not establish—an imbalance in PR isoform abundance.

Our data demonstrated increased total PR (PR-A/B) staining relative to PR-B staining in the endometrium of patients with endometriosis across the sampled menstrual-cycle phases. This pattern is compatible with, but does not directly demonstrate, increased relative PR-A abundance. Direct comparison of staining intensity across two antibodies is limited by potential differences in antibody affinity, epitope accessibility, and signal dynamic range. Accordingly, neither the total PR-versus-PR-B comparison nor the PR-B:total PR relative-staining index should be interpreted as a validated PR-A:PR-B ratio. Prior studies using complementary protein or transcript-based methods have reported altered PR isoform balance in endometriosis [19,21,33,34,35], supporting the rationale for future validation with PR-A-specific protein detection, isoform-specific qPCR, RNAscope, or other quantitative approaches.

There were no significant differences in PR-B staining intensity or localization between patients with and without endometriosis. Likewise, pSer344/345-PR staining was not significantly different between groups (*p* = 0.28). The observed numerical increase in the endometriosis group is therefore hypothesis-generating only and does not establish a role for PR phosphorylation in disease development. We selected Ser344/345 because prior uterine studies using this antibody demonstrated robust detection and functional effects of phosphorylation at this site in myometrial cells [31]. However, only this single phosphorylation site was examined; future studies should evaluate additional sites, including PR-B-specific Ser81, Ser102, and Ser162, together with quantitative isoform-specific assays.

In patients with endometriosis, PR immunostaining was also observed in ectopic peritoneal lesions, with lower overall staining intensity than in paired eutopic endometrium. The PR-B:total PR relative-staining index in eutopic endometrium was not statistically different between groups. These findings are descriptive and should not be interpreted as evidence of a specific PR-A:PR-B ratio or as establishing a causal mechanism.

Differences between eutopic and ectopic tissue may reflect lesion composition, cellular heterogeneity, menstrual-cycle effects, or disease phenotype. Prior studies have associated altered PR isoform balance with endometriosis phenotype and recurrence [33,34,35], but the present small cohort was not designed to test lesion aggressiveness or invasion mechanisms. Comparisons with breast-cancer models can provide biologic context for PR isoform function, but those mechanisms cannot be directly extrapolated to endometriosis.

Site-specific phosphorylation can modify PR activity in experimental systems, including breast cancer and uterine myometrial models [29,30,31,36]. These data support phosphorylation as a plausible regulatory mechanism but do not demonstrate that pSer344/345-PR drives endometriosis in the present cohort. The nonsignificant pSer344/345-PR finding should instead motivate larger studies incorporating multiple phosphorylation sites and quantitative molecular validation.

Inflammation is another plausible modifier of progesterone signaling. Endometriosis is characterized by local inflammatory signaling, and proinflammatory stimuli can alter PR expression or function in uterine cells [27,37]. Population studies have also used blood-cell-derived indices such as neutrophil-to-lymphocyte ratio and systemic immune-inflammation index as accessible markers of systemic inflammatory status [38]. However, such systemic biomarkers are not equivalent to local endometrial or peritoneal inflammation, and the present study did not measure inflammatory biomarkers. Thus, any proposed feedback between inflammation and altered PR signaling remains mechanistic hypothesis rather than a conclusion supported directly by these data.

Strengths of this study include strict eligibility criteria, histologic confirmation of endometriosis, synchronous collection of eutopic and peritoneal tissues, and blinded IHC assessment. The study also provides preliminary characterization of pSer344/345-PR staining in endometrial tissue. However, the original sample-size estimate was derived from quantitative immunoblot data rather than the ordinal 0–3 IHC outcome used here. The study should therefore be considered exploratory rather than confirmatory.

This study has several important limitations. The total cohort was small (*n* = 10 per group), and menstrual-phase subgroup comparisons included only 4–6 participants per group, limiting precision and statistical power. Menstrual stage was determined from the last menstrual period and confirmed histologically, but surgical scheduling prevented uniform sampling at a single cycle time point. The endometriosis group was significantly younger than the control group; with this sample size, age could not be robustly controlled and may confound receptor-expression comparisons. All control participants had infertility, which may itself be associated with altered endometrial biology and limits generalizability to fertile controls. Detailed disease staging and lesion phenotype were not uniformly captured beyond documentation that two patients had deep endometriosis, and control surgical indications included myomectomy and diagnostic laparoscopy for unexplained infertility. Participants had received no hormonal treatment affecting fertility or menstrual cycles for at least three months before surgery, but prior treatment history and infertility etiology were not systematically characterized for all participants. Methodologically, IHC provides an ordinal semi-quantitative score, direct comparison of signal intensity between different antibodies is imperfect, and the PR-B:total PR index has not been technically validated as a PR-A:PR-B ratio. Additionally, the exploratory PR-B:total PR relative-staining index was evaluated using pooled data rather than separately by menstrual-cycle phase and cellular compartment. Given known variation in PR expression across these factors, pooling may obscure phase- or compartment-specific differences. The small subgroup sizes and absence of patient-level stratified ratio data precluded meaningful further analysis in the present study. The phase- and compartment-specific analyses involved multiple unadjusted comparisons and should be considered hypothesis-generating. Finally, paired eutopic and ectopic specimens require statistical methods that account for within-patient correlation in confirmatory analyses. Larger, age-matched studies using fertile and infertile comparator groups, prespecified paired/nonparametric analyses, and quantitative isoform-specific methods are needed.

## 5. Conclusions

In this preliminary exploratory study, eutopic endometrium from patients with endometriosis demonstrated increased total PR (PR-A/B) staining relative to PR-B staining compared with infertile controls without endometriosis, and a similar qualitative pattern was observed in ectopic lesions. Because PR-A was not measured directly and staining generated by different antibodies is not directly equivalent, these findings provide indirect evidence of altered relative PR isoform abundance rather than proof of a PR-A-dominant state or a changed PR-A:PR-B ratio. pSer344/345-PR staining did not differ significantly between groups, so no conclusion can be drawn regarding a causal role for phosphorylation in endometriosis. These hypothesis-generating findings support future studies using larger age-matched cohorts, fertile comparator groups, paired statistical analyses, and quantitative isoform-specific and phospho-site-specific methods. Selective modulation of PR signaling may be a direction for future mechanistic and therapeutic research, but therapeutic efficacy is not established by the present data.

## Figures and Tables

**Table 1:** Patient characteristics for the endometriosis (*n* = 10) and no endometriosis (*n* = 10) groups.

Patient Characteristics	Endometriosis (*n* = 10)	No Endometriosis (*n* = 10)
Age	31.1 (SD 4.15)	35.7 (SD 3.92)
BMI	30.146 (SD 4.34)	31.802 (SD 6.78)
Ethnicity	70% white, 20% black, 10% Asian	70% white, 30% black

**Table 2:** Exploratory comparison of total PR (PR-A/B), PR-B, and pSer344/345-PR staining intensity in glandular and stromal regions of eutopic endometrium, stratified by menstrual-cycle phase. PR = progesterone receptor. Values are mean ± SD. Subgroup sizes are small (*n* = 4–6), and *p*-values are unadjusted for multiple comparisons; results should therefore be interpreted as hypothesis-generating.

	Proliferative Phase			Secretory Phase		
	Endometriosis (*n* = 5)	No Endometriosis (*n* = 4)	*p*-Value	Endometriosis (*n* = 5)	No Endometriosis (*n* = 6)	*p*-Value
Total PR (PR-A/B)						
Glandular	2.80 ± 0.27	1.88 ± 0.25	0.008	2.80 ± 0.45	1.67 ± 0.88	0.046
Stromal	2.60 ± 0.42	1.75 ± 0.50	0.040	2.50 ± 0.50	1.50 ± 0.63	0.039
PR-B						
Glandular	1.90 ± 0.65	1.38 ± 1.11	0.532	1.90 ± 1.08	1.75 ± 1.03	0.844
Stromal	1.90 ± 0.42	1.25 ± 0.29	0.079	1.60 ± 1.08	1.58 ± 0.86	0.836
pSer344/345-PR						
Glandular	2.10 ± 1.25	2.00 ± 0.82	0.738	2.20 ± 0.76	1.42 ± 1.11	0.294
Stromal	2.20 ± 0.76	1.88 ± 0.85	0.635	2.00 ± 0.79	1.42 ± 0.92	0.346

## Data Availability

The subjects in this trial have not concomitantly been involved in other trials. Data regarding any of the subjects in the study has not been previously published unless specified. Data will be made available to the editors of the journal for review or query upon request pre and/or post publication. The appropriate checklist for this study design was followed.
